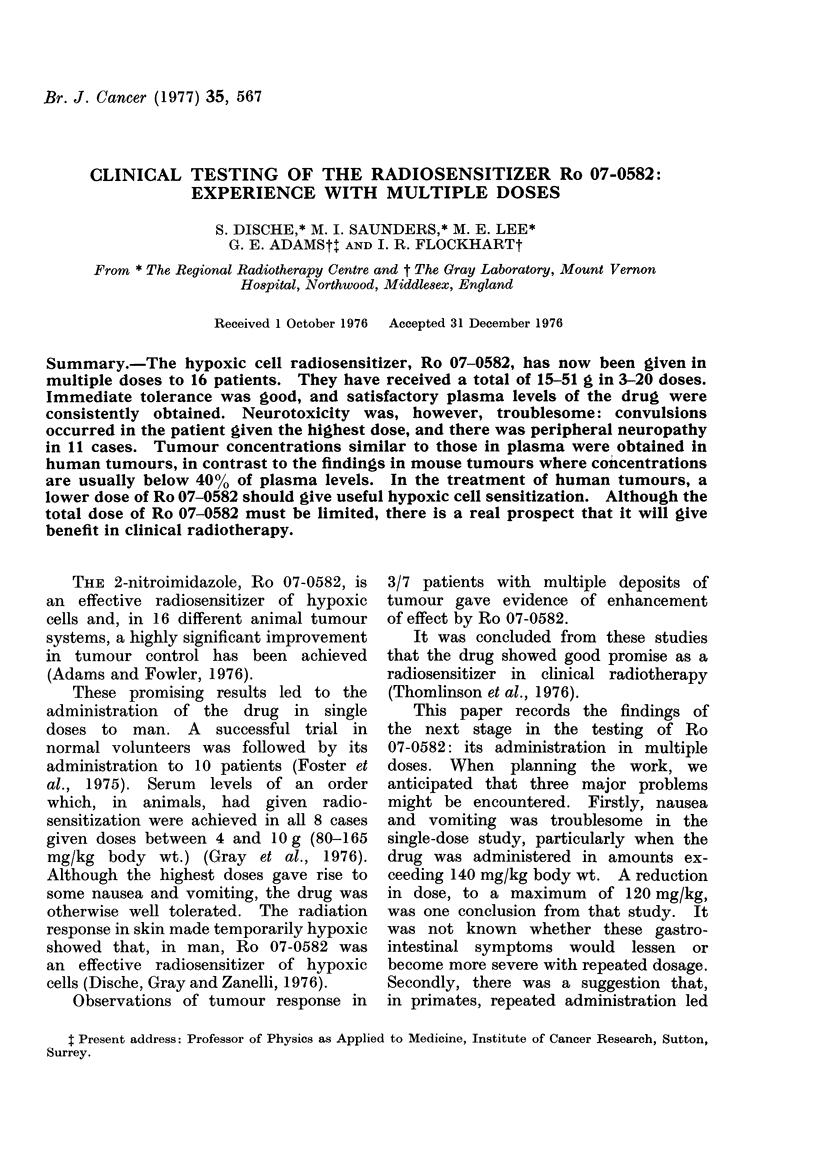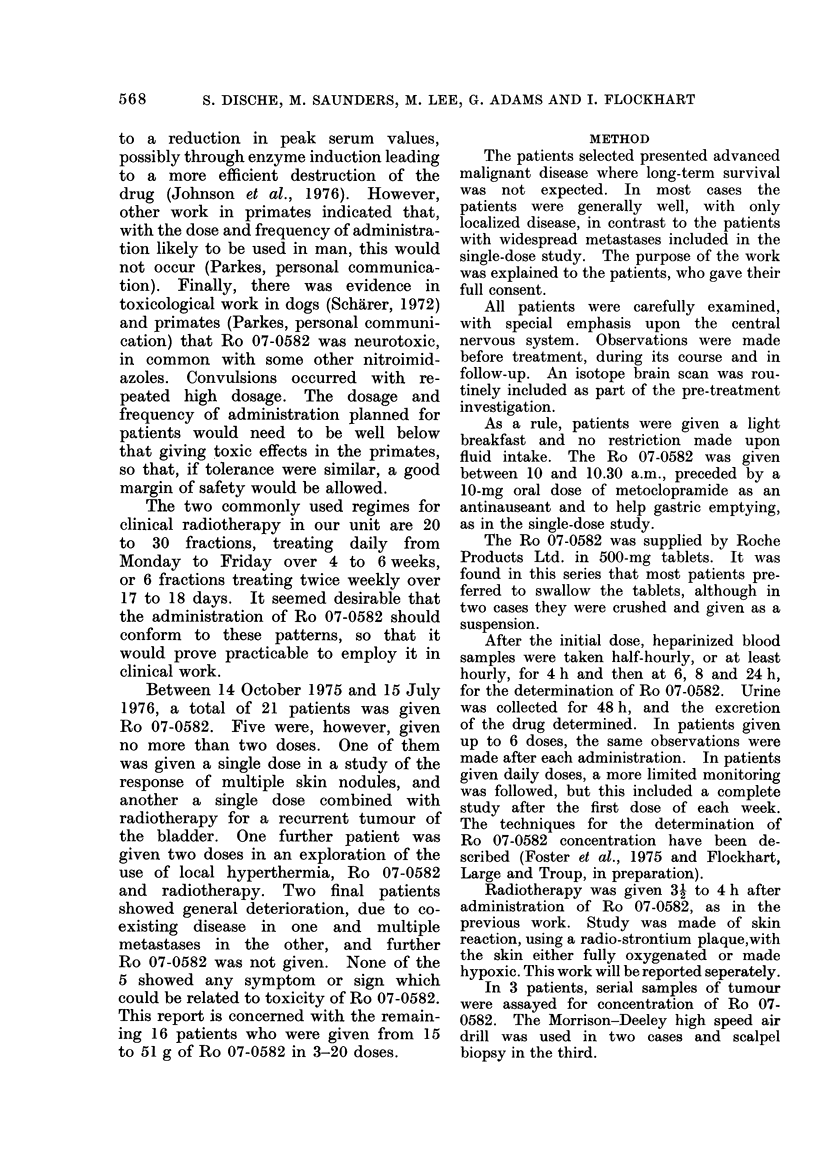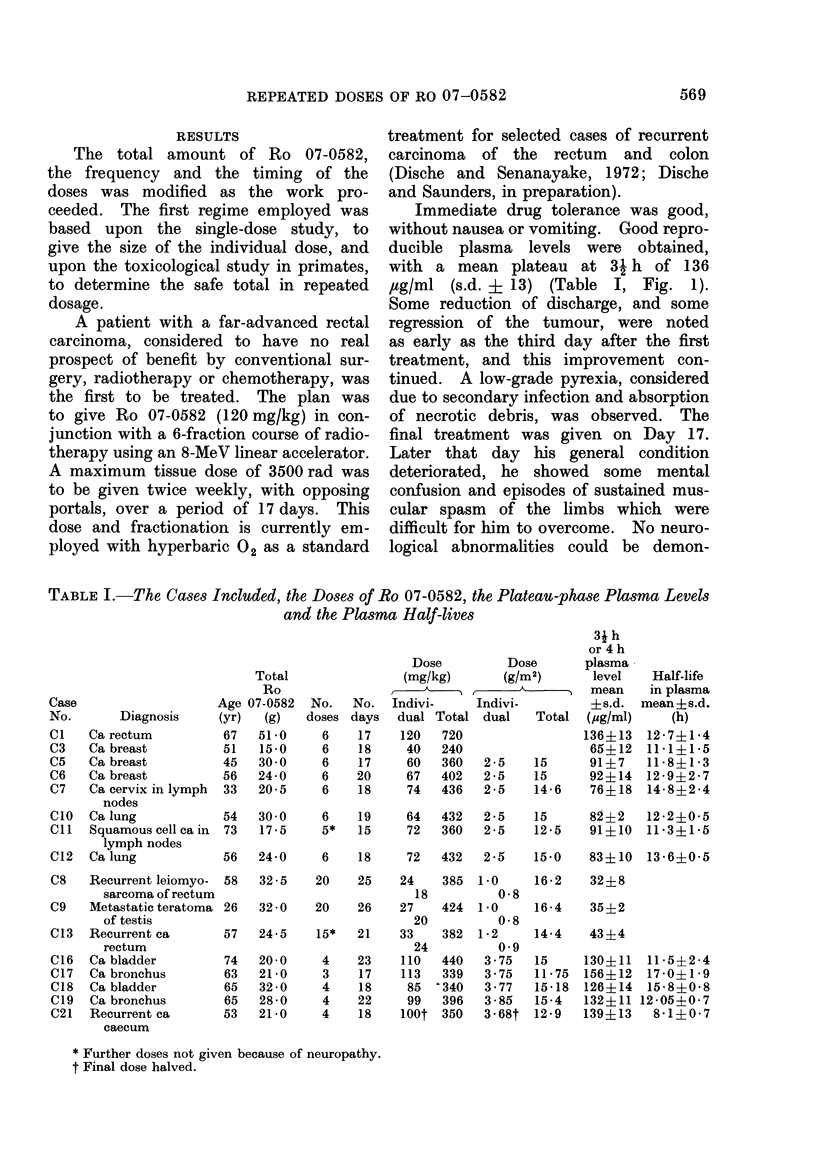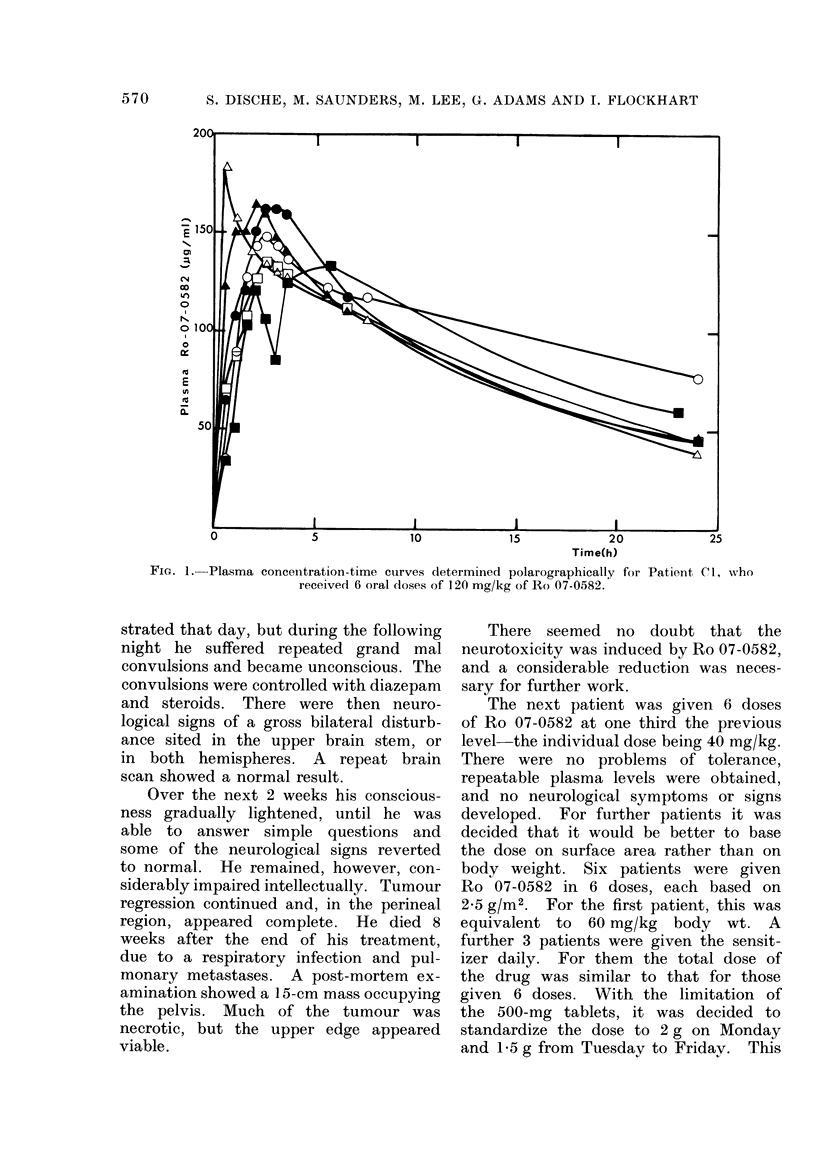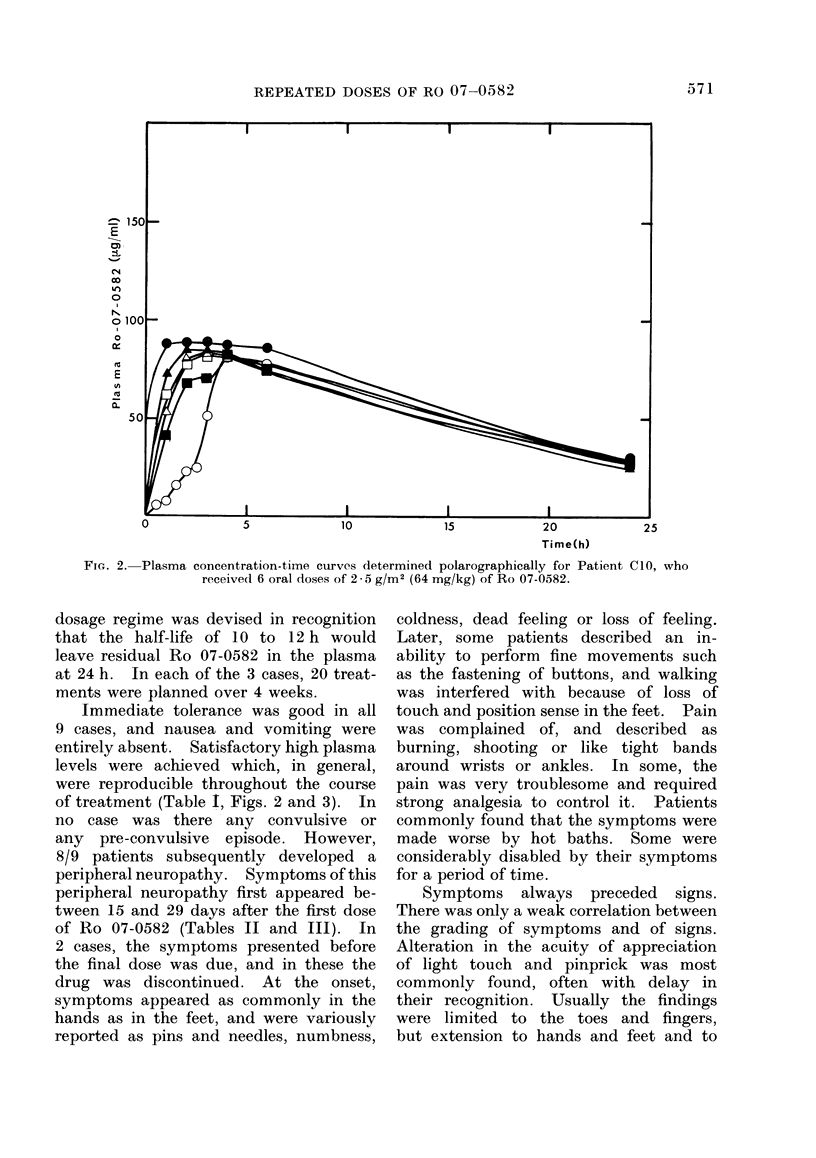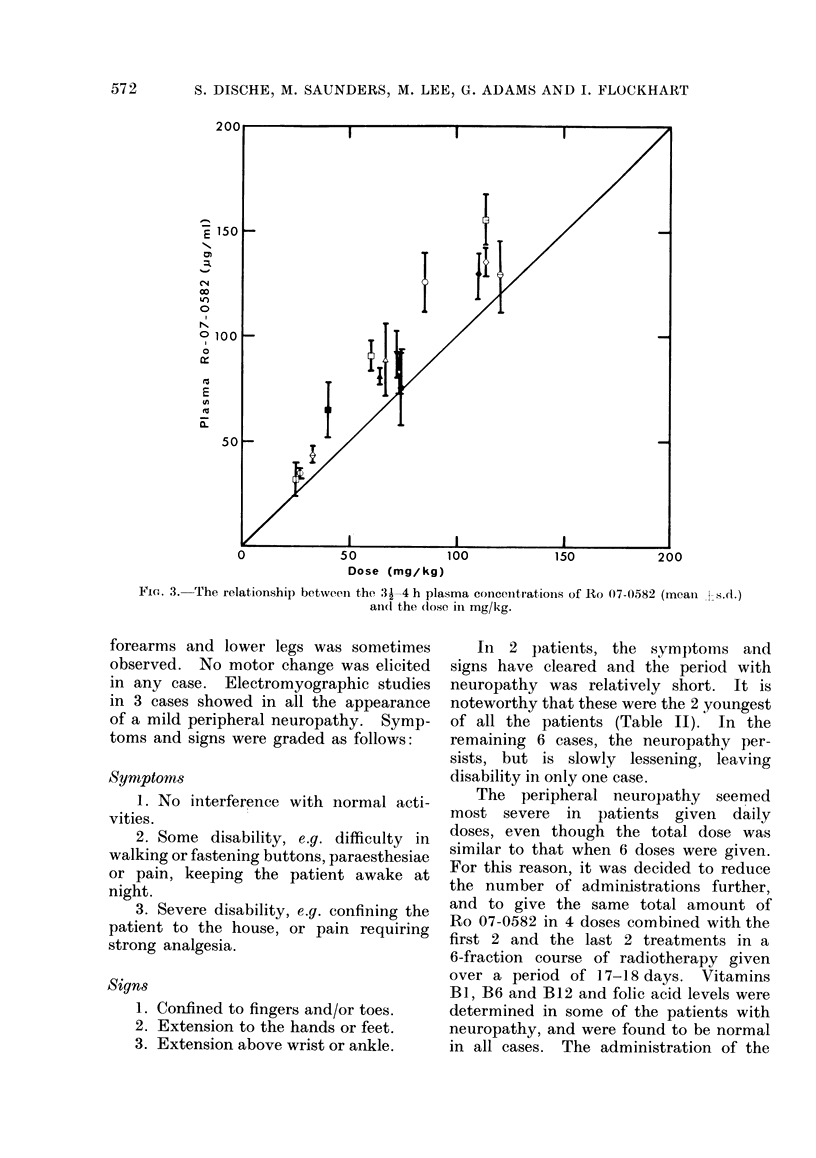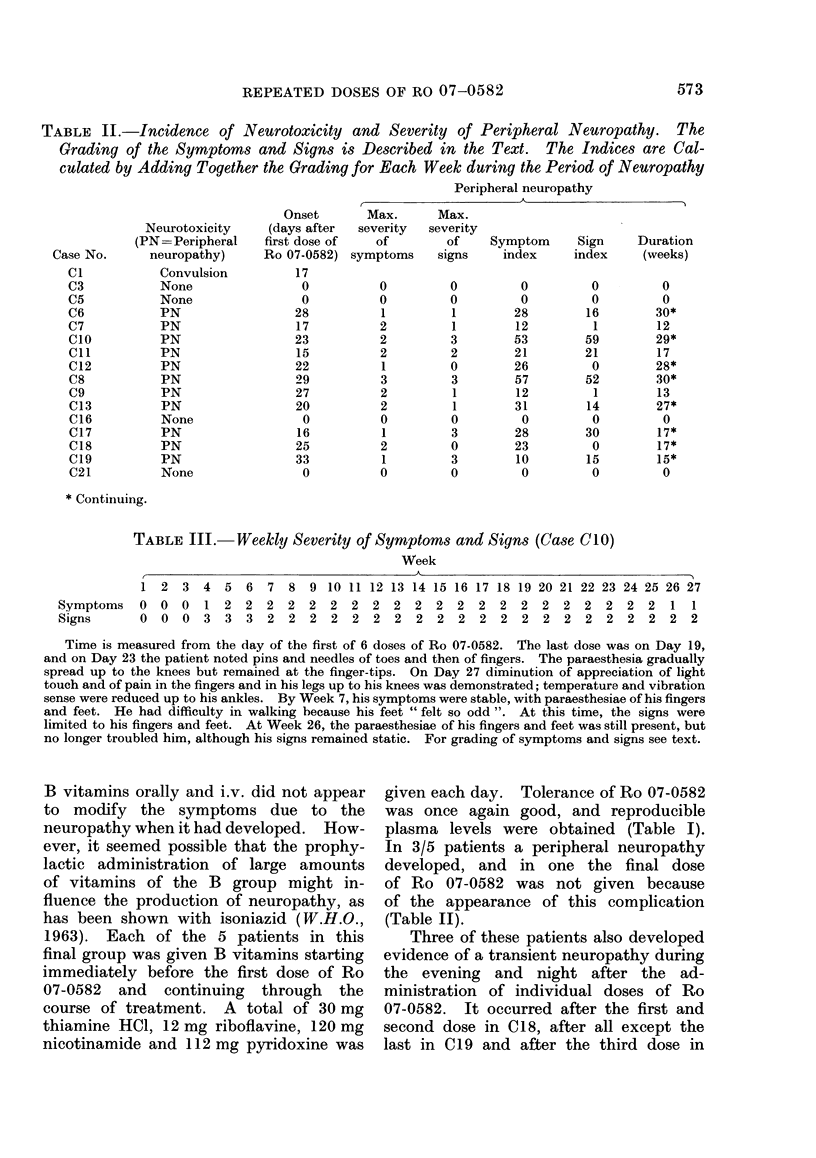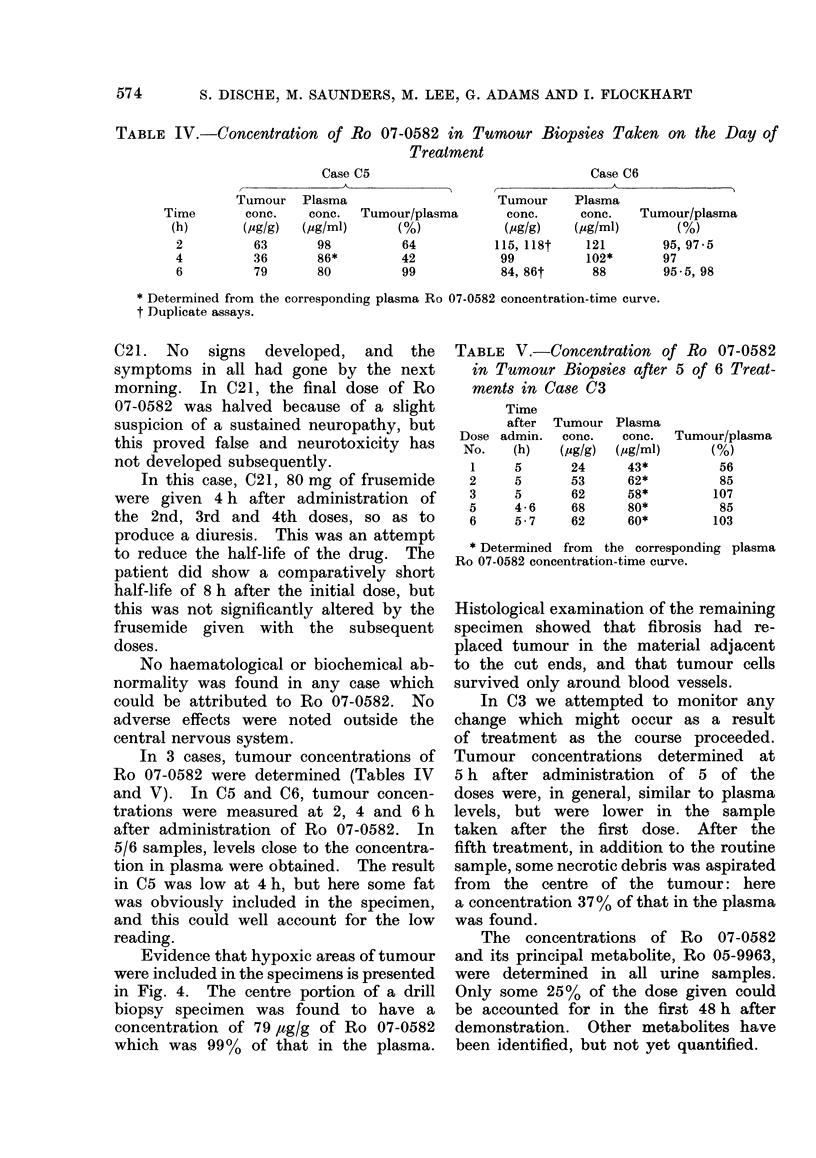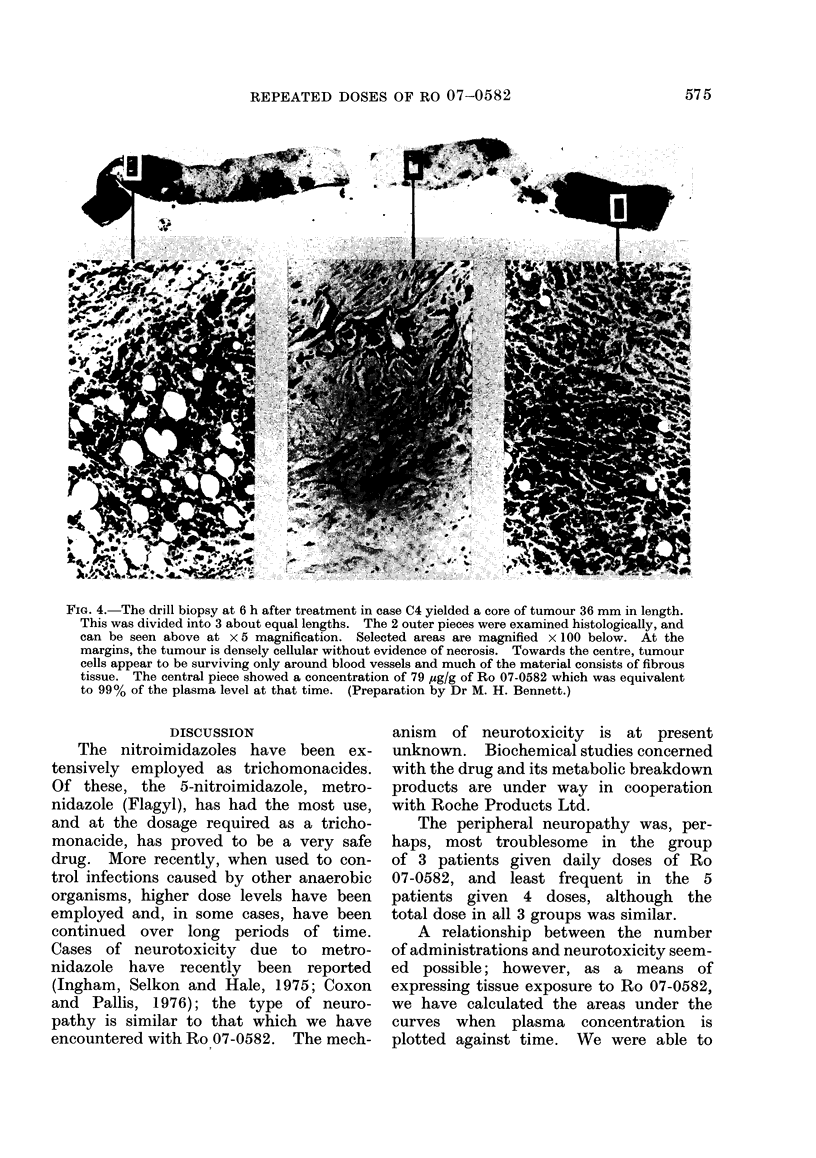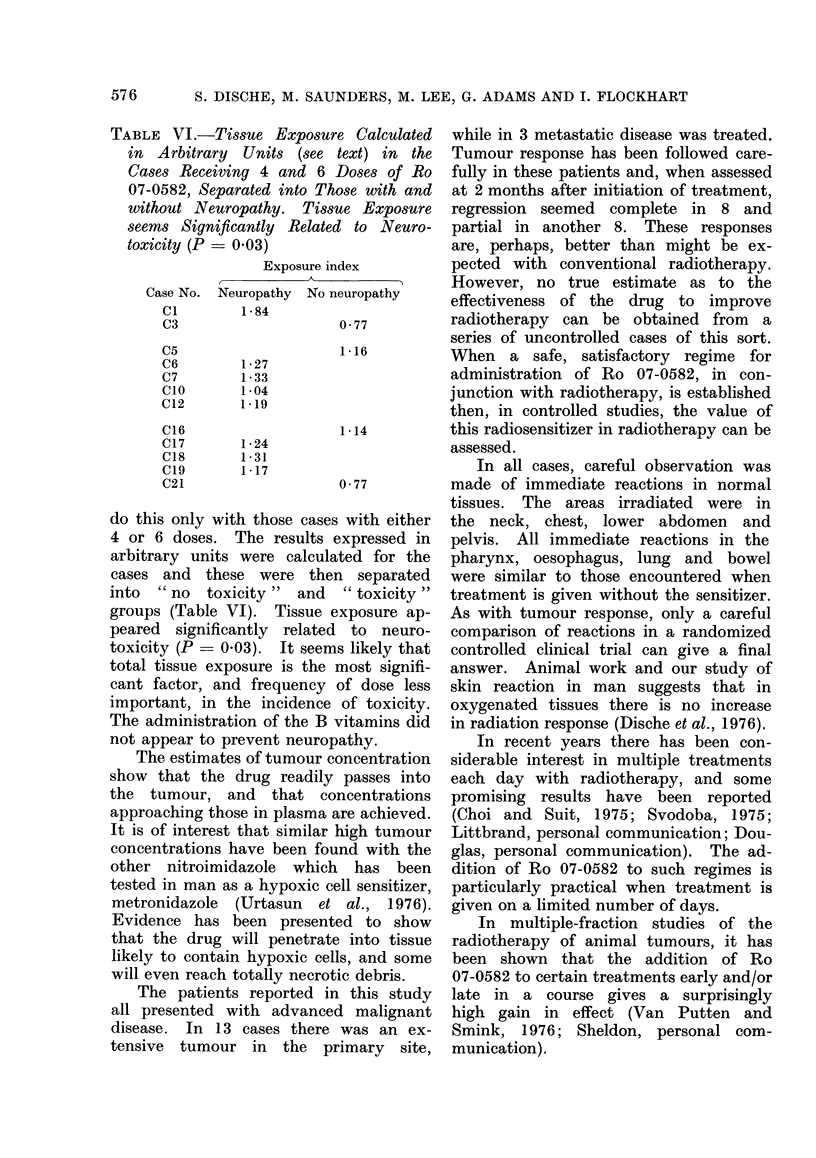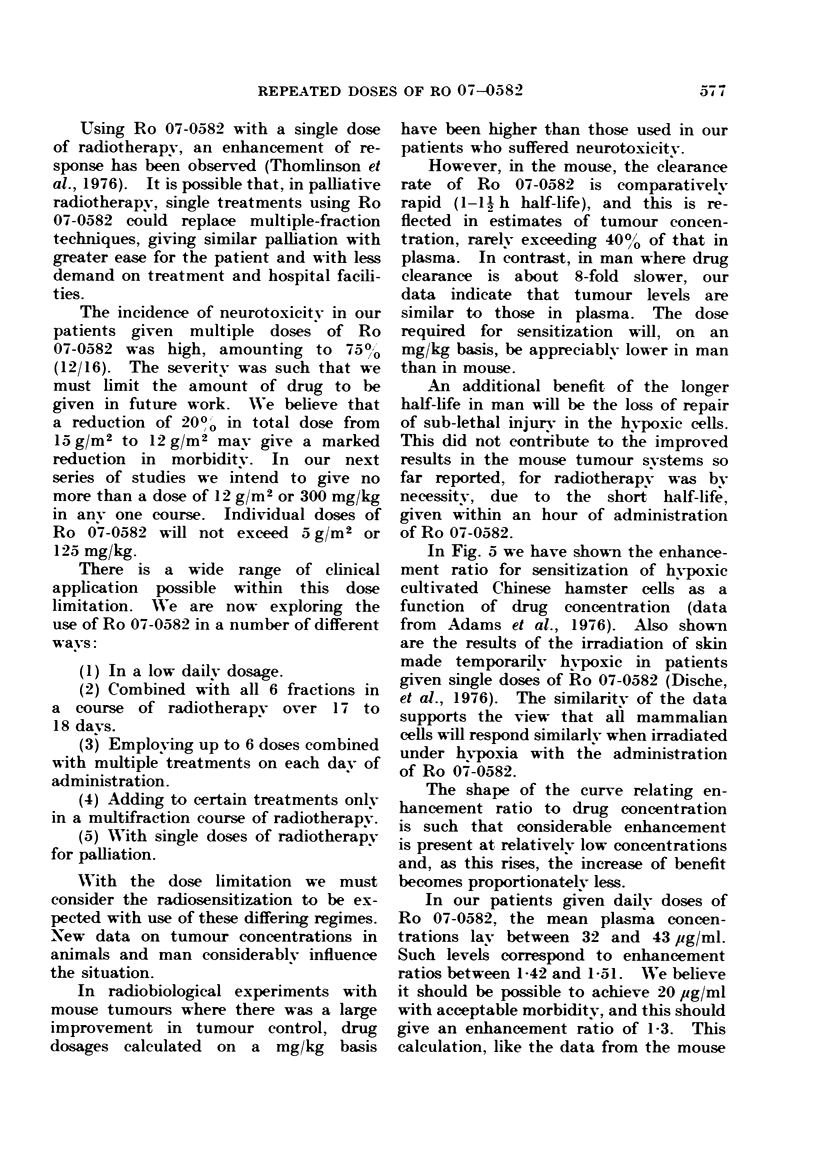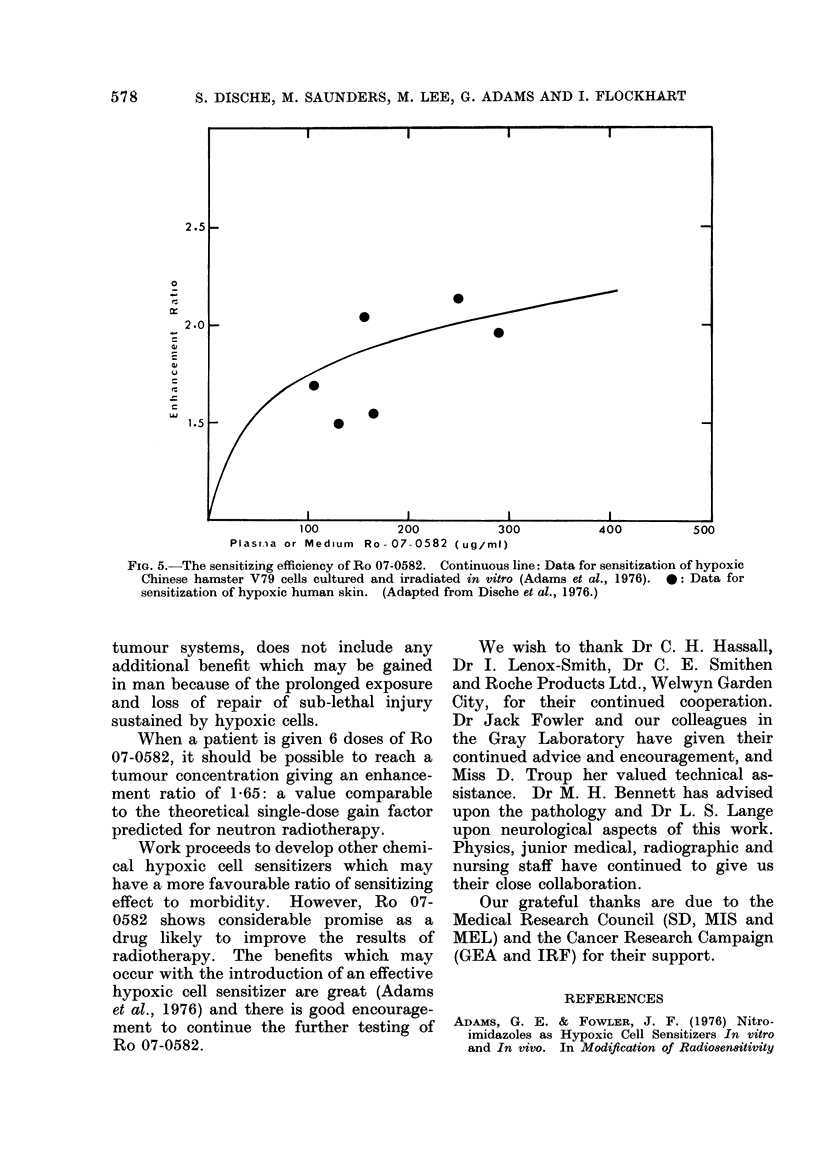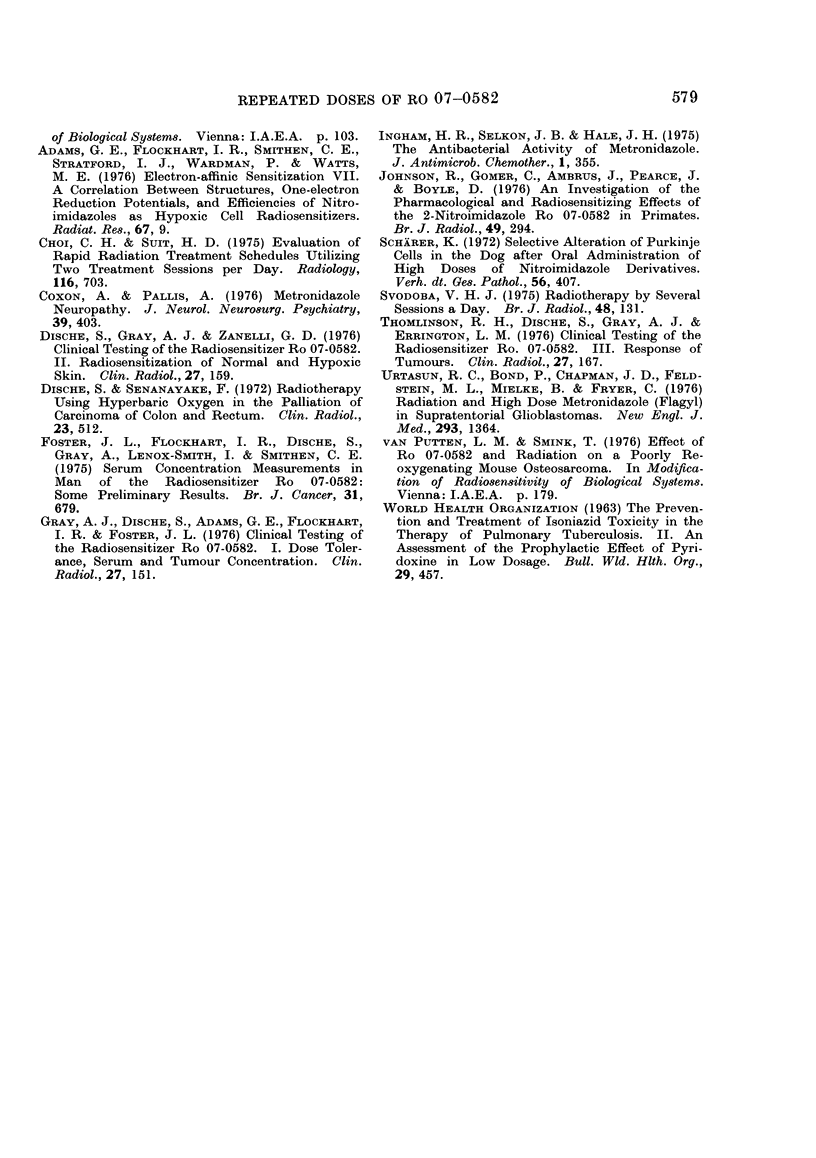# Clinical testing of the radiosensitizer Ro 07-0582: experience with multiple doses.

**DOI:** 10.1038/bjc.1977.90

**Published:** 1977-05

**Authors:** S. Dische, M. I. Saunders, M. E. Lee, G. E. Adams, I. R. Flockhart

## Abstract

**Images:**


*Br. J. Cancer* (1977) **35**, 567

### CLINICAL TESTING OF THE RADIOSENSITIZER Ro 07-0582: EXPERIENCE WITH MULTIPLE DOSES

S. DISCHE,* M. I. SAUNDERS,* M. E. LEE*
G. E. ADAMS†‡ AND I. R. FLOCKHART†

*From * The Regional Radiotherapy Centre and † The Gray Laboratory, Mount Vernon Hospital, Northwood, Middlesex, England*

Received 1 October 1976 Accepted 31 December 1976

**Summary.—The hypoxic cell radiosensitizer, Ro 07–0582, has now been given in multiple doses to 16 patients. They have received a total of 15–51 g in 3–20 doses. Immediate tolerance was good, and satisfactory plasma levels of the drug were consistently obtained. Neurotoxicity was, however, troublesome: convulsions occurred in the patient given the highest dose, and there was peripheral neuropathy in 11 cases. Tumour concentrations similar to those in plasma were obtained in human tumours, in contrast to the findings in mouse tumours where concentrations are usually below 40% of plasma levels. In the treatment of human tumours, a lower dose of Ro 07–0582 should give useful hypoxic cell sensitization. Although the total dose of Ro 07–0582 must be limited, there is a real prospect that it will give benefit in clinical radiotherapy.**

THE 2-nitroimidazole, Ro 07-0582, is an effective radiosensitizer of hypoxic cells and, in 16 different animal tumour systems, a highly significant improvement in tumour control has been achieved (Adams and Fowler, 1976).

These promising results led to the administration of the drug in single doses to man. A successful trial in normal volunteers was followed by its administration to 10 patients (Foster *et al.*, 1975). Serum levels of an order which, in animals, had given radiosensitization were achieved in all 8 cases given doses between 4 and 10 g (80–165 mg/kg body wt.) (Gray *et al.*, 1976). Although the highest doses gave rise to some nausea and vomiting, the drug was otherwise well tolerated. The radiation response in skin made temporarily hypoxic showed that, in man, Ro 07-0582 was an effective radiosensitizer of hypoxic cells (Dische, Gray and Zanelli, 1976).

Observations of tumour response in 3/7 patients with multiple deposits of tumour gave evidence of enhancement of effect by Ro 07-0582.

It was concluded from these studies that the drug showed good promise as a radiosensitizer in clinical radiotherapy (Thomlinson *et al.*, 1976).

This paper records the findings of the next stage in the testing of Ro 07-0582: its administration in multiple doses. When planning the work, we anticipated that three major problems might be encountered. Firstly, nausea and vomiting was troublesome in the single-dose study, particularly when the drug was administered in amounts exceeding 140 mg/kg body wt. A reduction in dose, to a maximum of 120 mg/kg, was one conclusion from that study. It was not known whether these gastrointestinal symptoms would lessen or become more severe with repeated dosage. Secondly, there was a suggestion that, in primates, repeated administration led

‡ Present address: Professor of Physics as Applied to Medicine, Institute of Cancer Research, Sutton, Surrey.

to a reduction in peak serum values, possibly through enzyme induction leading to a more efficient destruction of the drug (Johnson *et al.*, 1976). However, other work in primates indicated that, with the dose and frequency of administration likely to be used in man, this would not occur (Parkes, personal communication). Finally, there was evidence in toxicological work in dogs (Schärer, 1972) and primates (Parkes, personal communication) that Ro 07-0582 was neurotoxic, in common with some other nitroimidazoles. Convulsions occurred with repeated high dosage. The dosage and frequency of administration planned for patients would need to be well below that giving toxic effects in the primates, so that, if tolerance were similar, a good margin of safety would be allowed.

The two commonly used regimes for clinical radiotherapy in our unit are 20 to 30 fractions, treating daily from Monday to Friday over 4 to 6 weeks, or 6 fractions treating twice weekly over 17 to 18 days. It seemed desirable that the administration of Ro 07-0582 should conform to these patterns, so that it would prove practicable to employ it in clinical work.

Between 14 October 1975 and 15 July 1976, a total of 21 patients was given Ro 07-0582. Five were, however, given no more than two doses. One of them was given a single dose in a study of the response of multiple skin nodules, and another a single dose combined with radiotherapy for a recurrent tumour of the bladder. One further patient was given two doses in an exploration of the use of local hyperthermia, Ro 07-0582 and radiotherapy. Two final patients showed general deterioration, due to coexisting disease in one and multiple metastases in the other, and further Ro 07-0582 was not given. None of the 5 showed any symptom or sign which could be related to toxicity of Ro 07-0582. This report is concerned with the remaining 16 patients who were given from 15 to 51 g of Ro 07-0582 in 3–20 doses.

### METHOD

The patients selected presented advanced malignant disease where long-term survival was not expected. In most cases the patients were generally well, with only localized disease, in contrast to the patients with widespread metastases included in the single-dose study. The purpose of the work was explained to the patients, who gave their full consent.

All patients were carefully examined, with special emphasis upon the central nervous system. Observations were made before treatment, during its course and in follow-up. An isotope brain scan was routinely included as part of the pre-treatment investigation.

As a rule, patients were given a light breakfast and no restriction made upon fluid intake. The Ro 07-0582 was given between 10 and 10.30 a.m., preceded by a 10-mg oral dose of metoclopramide as an antinauseant and to help gastric emptying, as in the single-dose study.

The Ro 07-0582 was supplied by Roche Products Ltd. in 500-mg tablets. It was found in this series that most patients preferred to swallow the tablets, although in two cases they were crushed and given as a suspension.

After the initial dose, heparinized blood samples were taken half-hourly, or at least hourly, for 4 h and then at 6, 8 and 24 h, for the determination of Ro 07-0582. Urine was collected for 48 h, and the excretion of the drug determined. In patients given up to 6 doses, the same observations were made after each administration. In patients given daily doses, a more limited monitoring was followed, but this included a complete study after the first dose of each week. The techniques for the determination of Ro 07-0582 concentration have been described (Foster *et al.*, 1975 and Flockhart, Large and Troup, in preparation).

Radiotherapy was given $3\frac{1}{2}$ to 4 h after administration of Ro 07-0582, as in the previous work. Study was made of skin reaction, using a radio-strontium plaque, with the skin either fully oxygenated or made hypoxic. This work will be reported seperately.

In 3 patients, serial samples of tumour were assayed for concentration of Ro 07-0582. The Morrison–Deeley high speed air drill was used in two cases and scalpel biopsy in the third.

### RESULTS

The total amount of Ro 07-0582, the frequency and the timing of the doses was modified as the work proceeded. The first regime employed was based upon the single-dose study, to give the size of the individual dose, and upon the toxicological study in primates, to determine the safe total in repeated dosage.

A patient with a far-advanced rectal carcinoma, considered to have no real prospect of benefit by conventional surgery, radiotherapy or chemotherapy, was the first to be treated. The plan was to give Ro 07-0582 (120 mg/kg) in conjunction with a 6-fraction course of radiotherapy using an 8-MeV linear accelerator. A maximum tissue dose of 3500 rad was to be given twice weekly, with opposing portals, over a period of 17 days. This dose and fractionation is currently employed with hyperbaric $O_2$ as a standard treatment for selected cases of recurrent carcinoma of the rectum and colon (Dische and Senanayake, 1972; Dische and Saunders, in preparation).

Immediate drug tolerance was good, without nausea or vomiting. Good reproducible plasma levels were obtained, with a mean plateau at 3½ h of 136 μg/ml (s.d. ± 13) (Table I, Fig. 1). Some reduction of discharge, and some regression of the tumour, were noted as early as the third day after the first treatment, and this improvement continued. A low-grade pyrexia, considered due to secondary infection and absorption of necrotic debris, was observed. The final treatment was given on Day 17. Later that day his general condition deteriorated, he showed some mental confusion and episodes of sustained muscular spasm of the limbs which were difficult for him to overcome. No neurological abnormalities could be demon-

TABLE I.—*The Cases Included, the Doses of Ro 07-0582, the Plateau-phase Plasma Levels and the Plasma Half-lives*

| Case No. | Diagnosis | Age (yr) | Total Ro 07-0582 (g) | No. doses | No. days | Dose (mg/kg) Individual | Dose (mg/kg) Total | Dose (g/m²) Individual | Dose (g/m²) Total | 3½ h or 4 h plasma level mean ±s.d. (μg/ml) | Half-life in plasma mean±s.d. (h) |
|---|---|---|---|---|---|---|---|---|---|---|---|
| C1 | Ca rectum | 67 | 51·0 | 6 | 17 | 120 | 720 | | | 136±13 | 12·7±1·4 |
| C3 | Ca breast | 51 | 15·0 | 6 | 18 | 40 | 240 | | | 65±12 | 11·1±1·5 |
| C5 | Ca breast | 45 | 30·0 | 6 | 17 | 60 | 360 | 2·5 | 15 | 91±7 | 11·8±1·3 |
| C6 | Ca breast | 56 | 24·0 | 6 | 20 | 67 | 402 | 2·5 | 15 | 92±14 | 12·9±2·7 |
| C7 | Ca cervix in lymph nodes | 33 | 20·5 | 6 | 18 | 74 | 436 | 2·5 | 14·6 | 76±18 | 14·8±2·4 |
| C10 | Ca lung | 54 | 30·0 | 6 | 19 | 64 | 432 | 2·5 | 15 | 82±2 | 12·2±0·5 |
| C11 | Squamous cell ca in lymph nodes | 73 | 17·5 | 5* | 15 | 72 | 360 | 2·5 | 12·5 | 91±10 | 11·3±1·5 |
| C12 | Ca lung | 56 | 24·0 | 6 | 18 | 72 | 432 | 2·5 | 15·0 | 83±10 | 13·6±0·5 |
| C8 | Recurrent leiomyosarcoma of rectum | 58 | 32·5 | 20 | 25 | 24<br>18 | 385 | 1·0<br>0·8 | 16·2 | 32±8 | |
| C9 | Metastatic teratoma of testis | 26 | 32·0 | 20 | 26 | 27<br>20 | 424 | 1·0<br>0·8 | 16·4 | 35±2 | |
| C13 | Recurrent ca rectum | 57 | 24·5 | 15* | 21 | 33<br>24 | 382 | 1·2<br>0·9 | 14·4 | 43±4 | |
| C16 | Ca bladder | 74 | 20·0 | 4 | 23 | 110 | 440 | 3·75 | 15 | 130±11 | 11·5±2·4 |
| C17 | Ca bronchus | 63 | 21·0 | 3 | 17 | 113 | 339 | 3·75 | 11·75 | 156±12 | 17·0±1·9 |
| C18 | Ca bladder | 65 | 32·0 | 4 | 18 | 85 | 340 | 3·77 | 15·18 | 126±14 | 15·8±0·8 |
| C19 | Ca bronchus | 65 | 28·0 | 4 | 22 | 99 | 396 | 3·85 | 15·4 | 132±11 | 12·05±0·7 |
| C21 | Recurrent ca caecum | 53 | 21·0 | 4 | 18 | 100† | 350 | 3·68† | 12·9 | 139±13 | 8·1±0·7 |

* Further doses not given because of neuropathy.
† Final dose halved.

FIG. 1.—Plasma concentration-time curves determined polarographically for Patient C1, who received 6 oral doses of 120 mg/kg of Ro 07-0582.

strated that day, but during the following night he suffered repeated grand mal convulsions and became unconscious. The convulsions were controlled with diazepam and steroids. There were then neurological signs of a gross bilateral disturbance sited in the upper brain stem, or in both hemispheres. A repeat brain scan showed a normal result.

Over the next 2 weeks his consciousness gradually lightened, until he was able to answer simple questions and some of the neurological signs reverted to normal. He remained, however, considerably impaired intellectually. Tumour regression continued and, in the perineal region, appeared complete. He died 8 weeks after the end of his treatment, due to a respiratory infection and pulmonary metastases. A post-mortem examination showed a 15-cm mass occupying the pelvis. Much of the tumour was necrotic, but the upper edge appeared viable.

There seemed no doubt that the neurotoxicity was induced by Ro 07-0582, and a considerable reduction was necessary for further work.

The next patient was given 6 doses of Ro 07-0582 at one third the previous level—the individual dose being 40 mg/kg. There were no problems of tolerance, repeatable plasma levels were obtained, and no neurological symptoms or signs developed. For further patients it was decided that it would be better to base the dose on surface area rather than on body weight. Six patients were given Ro 07-0582 in 6 doses, each based on 2·5 g/m$^2$. For the first patient, this was equivalent to 60 mg/kg body wt. A further 3 patients were given the sensitizer daily. For them the total dose of the drug was similar to that for those given 6 doses. With the limitation of the 500-mg tablets, it was decided to standardize the dose to 2 g on Monday and 1·5 g from Tuesday to Friday. This

FIG. 2.—Plasma concentration-time curves determined polarographically for Patient C10, who received 6 oral doses of 2·5 g/m² (64 mg/kg) of Ro 07-0582.

dosage regime was devised in recognition that the half-life of 10 to 12 h would leave residual Ro 07-0582 in the plasma at 24 h. In each of the 3 cases, 20 treatments were planned over 4 weeks.

Immediate tolerance was good in all 9 cases, and nausea and vomiting were entirely absent. Satisfactory high plasma levels were achieved which, in general, were reproducible throughout the course of treatment (Table I, Figs. 2 and 3). In no case was there any convulsive or any pre-convulsive episode. However, 8/9 patients subsequently developed a peripheral neuropathy. Symptoms of this peripheral neuropathy first appeared between 15 and 29 days after the first dose of Ro 07-0582 (Tables II and III). In 2 cases, the symptoms presented before the final dose was due, and in these the drug was discontinued. At the onset, symptoms appeared as commonly in the hands as in the feet, and were variously reported as pins and needles, numbness, coldness, dead feeling or loss of feeling. Later, some patients described an inability to perform fine movements such as the fastening of buttons, and walking was interfered with because of loss of touch and position sense in the feet. Pain was complained of, and described as burning, shooting or like tight bands around wrists or ankles. In some, the pain was very troublesome and required strong analgesia to control it. Patients commonly found that the symptoms were made worse by hot baths. Some were considerably disabled by their symptoms for a period of time.

Symptoms always preceded signs. There was only a weak correlation between the grading of symptoms and of signs. Alteration in the acuity of appreciation of light touch and pinprick was most commonly found, often with delay in their recognition. Usually the findings were limited to the toes and fingers, but extension to hands and feet and to

FIG. 3.—The relationship between the $3\frac{1}{2}$–4 h plasma concentrations of Ro 07-0582 (mean ± s.d.) and the dose in mg/kg.

forearms and lower legs was sometimes observed. No motor change was elicited in any case. Electromyographic studies in 3 cases showed in all the appearance of a mild peripheral neuropathy. Symptoms and signs were graded as follows:

*Symptoms*

1. No interference with normal activities.
2. Some disability, *e.g.* difficulty in walking or fastening buttons, paraesthesiae or pain, keeping the patient awake at night.
3. Severe disability, *e.g.* confining the patient to the house, or pain requiring strong analgesia.

*Signs*

1. Confined to fingers and/or toes.
2. Extension to the hands or feet.
3. Extension above wrist or ankle.

In 2 patients, the symptoms and signs have cleared and the period with neuropathy was relatively short. It is noteworthy that these were the 2 youngest of all the patients (Table II). In the remaining 6 cases, the neuropathy persists, but is slowly lessening, leaving disability in only one case.

The peripheral neuropathy seemed most severe in patients given daily doses, even though the total dose was similar to that when 6 doses were given. For this reason, it was decided to reduce the number of administrations further, and to give the same total amount of Ro 07-0582 in 4 doses combined with the first 2 and the last 2 treatments in a 6-fraction course of radiotherapy given over a period of 17–18 days. Vitamins B1, B6 and B12 and folic acid levels were determined in some of the patients with neuropathy, and were found to be normal in all cases. The administration of the

TABLE II.—*Incidence of Neurotoxicity and Severity of Peripheral Neuropathy. The Grading of the Symptoms and Signs is Described in the Text. The Indices are Calculated by Adding Together the Grading for Each Week during the Period of Neuropathy*

| Case No. | Neurotoxicity (PN = Peripheral neuropathy) | Onset (days after first dose of Ro 07-0582) | Peripheral neuropathy: Max. severity of symptoms | Max. severity of signs | Symptom index | Sign index | Duration (weeks) |
|---|---|---|---|---|---|---|---|
| C1 | Convulsion | 17 | | | | | |
| C3 | None | 0 | 0 | 0 | 0 | 0 | 0 |
| C5 | None | 0 | 0 | 0 | 0 | 0 | 0 |
| C6 | PN | 28 | 1 | 1 | 28 | 16 | 30* |
| C7 | PN | 17 | 2 | 1 | 12 | 1 | 12 |
| C10 | PN | 23 | 2 | 3 | 53 | 59 | 29* |
| C11 | PN | 15 | 2 | 2 | 21 | 21 | 17 |
| C12 | PN | 22 | 1 | 0 | 26 | 0 | 28* |
| C8 | PN | 29 | 3 | 3 | 57 | 52 | 30* |
| C9 | PN | 27 | 2 | 1 | 12 | 1 | 13 |
| C13 | PN | 20 | 2 | 1 | 31 | 14 | 27* |
| C16 | None | 0 | 0 | 0 | 0 | 0 | 0 |
| C17 | PN | 16 | 1 | 3 | 28 | 30 | 17* |
| C18 | PN | 25 | 2 | 0 | 23 | 0 | 17* |
| C19 | PN | 33 | 1 | 3 | 10 | 15 | 15* |
| C21 | None | 0 | 0 | 0 | 0 | 0 | 0 |

* Continuing.

TABLE III.—*Weekly Severity of Symptoms and Signs (Case C10)*

| Week | 1 | 2 | 3 | 4 | 5 | 6 | 7 | 8 | 9 | 10 | 11 | 12 | 13 | 14 | 15 | 16 | 17 | 18 | 19 | 20 | 21 | 22 | 23 | 24 | 25 | 26 | 27 |
|---|---|---|---|---|---|---|---|---|---|---|---|---|---|---|---|---|---|---|---|---|---|---|---|---|---|---|---|
| Symptoms | 0 | 0 | 0 | 1 | 2 | 2 | 2 | 2 | 2 | 2 | 2 | 2 | 2 | 2 | 2 | 2 | 2 | 2 | 2 | 2 | 2 | 2 | 2 | 2 | 2 | 1 | 1 |
| Signs | 0 | 0 | 0 | 3 | 3 | 3 | 2 | 2 | 2 | 2 | 2 | 2 | 2 | 2 | 2 | 2 | 2 | 2 | 2 | 2 | 2 | 2 | 2 | 2 | 2 | 2 | 2 |

Time is measured from the day of the first of 6 doses of Ro 07-0582. The last dose was on Day 19, and on Day 23 the patient noted pins and needles of toes and then of fingers. The paraesthesia gradually spread up to the knees but remained at the finger-tips. On Day 27 diminution of appreciation of light touch and of pain in the fingers and in his legs up to his knees was demonstrated; temperature and vibration sense were reduced up to his ankles. By Week 7, his symptoms were stable, with paraesthesiae of his fingers and feet. He had difficulty in walking because his feet " felt so odd ". At this time, the signs were limited to his fingers and feet. At Week 26, the paraesthesiae of his fingers and feet was still present, but no longer troubled him, although his signs remained static. For grading of symptoms and signs see text.

B vitamins orally and i.v. did not appear to modify the symptoms due to the neuropathy when it had developed. However, it seemed possible that the prophylactic administration of large amounts of vitamins of the B group might influence the production of neuropathy, as has been shown with isoniazid (*W.H.O.*, 1963). Each of the 5 patients in this final group was given B vitamins starting immediately before the first dose of Ro 07-0582 and continuing through the course of treatment. A total of 30 mg thiamine HCl, 12 mg riboflavine, 120 mg nicotinamide and 112 mg pyridoxine was given each day. Tolerance of Ro 07-0582 was once again good, and reproducible plasma levels were obtained (Table I). In 3/5 patients a peripheral neuropathy developed, and in one the final dose of Ro 07-0582 was not given because of the appearance of this complication (Table II).

Three of these patients also developed evidence of a transient neuropathy during the evening and night after the administration of individual doses of Ro 07-0582. It occurred after the first and second dose in C18, after all except the last in C19 and after the third dose in

TABLE IV.—*Concentration of Ro* 07-0582 *in Tumour Biopsies Taken on the Day of Treatment*

| | Case C5 | | | Case C6 | | |
|---|---|---|---|---|---|---|
| Time (h) | Tumour conc. (μg/g) | Plasma conc. (μg/ml) | Tumour/plasma (%) | Tumour conc. (μg/g) | Plasma conc. (μg/ml) | Tumour/plasma (%) |
| 2 | 63 | 98 | 64 | 115, 118† | 121 | 95, 97·5 |
| 4 | 36 | 86* | 42 | 99 | 102* | 97 |
| 6 | 79 | 80 | 99 | 84, 86† | 88 | 95·5, 98 |

* Determined from the corresponding plasma Ro 07-0582 concentration-time curve.
† Duplicate assays.

C21. No signs developed, and the symptoms in all had gone by the next morning. In C21, the final dose of Ro 07-0582 was halved because of a slight suspicion of a sustained neuropathy, but this proved false and neurotoxicity has not developed subsequently.

In this case, C21, 80 mg of frusemide were given 4 h after administration of the 2nd, 3rd and 4th doses, so as to produce a diuresis. This was an attempt to reduce the half-life of the drug. The patient did show a comparatively short half-life of 8 h after the initial dose, but this was not significantly altered by the frusemide given with the subsequent doses.

No haematological or biochemical abnormality was found in any case which could be attributed to Ro 07-0582. No adverse effects were noted outside the central nervous system.

In 3 cases, tumour concentrations of Ro 07-0582 were determined (Tables IV and V). In C5 and C6, tumour concentrations were measured at 2, 4 and 6 h after administration of Ro 07-0582. In 5/6 samples, levels close to the concentration in plasma were obtained. The result in C5 was low at 4 h, but here some fat was obviously included in the specimen, and this could well account for the low reading.

Evidence that hypoxic areas of tumour were included in the specimens is presented in Fig. 4. The centre portion of a drill biopsy specimen was found to have a concentration of 79 μg/g of Ro 07-0582 which was 99% of that in the plasma.

TABLE V.—*Concentration of Ro* 07-0582 *in Tumour Biopsies after* 5 *of* 6 *Treatments in Case C*3

| Dose No. | Time after admin. (h) | Tumour conc. (μg/g) | Plasma conc. (μg/ml) | Tumour/plasma (%) |
|---|---|---|---|---|
| 1 | 5 | 24 | 43* | 56 |
| 2 | 5 | 53 | 62* | 85 |
| 3 | 5 | 62 | 58* | 107 |
| 5 | 4·6 | 68 | 80* | 85 |
| 6 | 5·7 | 62 | 60* | 103 |

* Determined from the corresponding plasma Ro 07-0582 concentration-time curve.

Histological examination of the remaining specimen showed that fibrosis had replaced tumour in the material adjacent to the cut ends, and that tumour cells survived only around blood vessels.

In C3 we attempted to monitor any change which might occur as a result of treatment as the course proceeded. Tumour concentrations determined at 5 h after administration of 5 of the doses were, in general, similar to plasma levels, but were lower in the sample taken after the first dose. After the fifth treatment, in addition to the routine sample, some necrotic debris was aspirated from the centre of the tumour: here a concentration 37% of that in the plasma was found.

The concentrations of Ro 07-0582 and its principal metabolite, Ro 05-9963, were determined in all urine samples. Only some 25% of the dose given could be accounted for in the first 48 h after demonstration. Other metabolites have been identified, but not yet quantified.

FIG. 4.—The drill biopsy at 6 h after treatment in case C4 yielded a core of tumour 36 mm in length. This was divided into 3 about equal lengths. The 2 outer pieces were examined histologically, and can be seen above at ×5 magnification. Selected areas are magnified ×100 below. At the margins, the tumour is densely cellular without evidence of necrosis. Towards the centre, tumour cells appear to be surviving only around blood vessels and much of the material consists of fibrous tissue. The central piece showed a concentration of 79 μg/g of Ro 07-0582 which was equivalent to 99% of the plasma level at that time. (Preparation by Dr M. H. Bennett.)

### DISCUSSION

The nitroimidazoles have been extensively employed as trichomonacides. Of these, the 5-nitroimidazole, metronidazole (Flagyl), has had the most use, and at the dosage required as a trichomonacide, has proved to be a very safe drug. More recently, when used to control infections caused by other anaerobic organisms, higher dose levels have been employed and, in some cases, have been continued over long periods of time. Cases of neurotoxicity due to metronidazole have recently been reported (Ingham, Selkon and Hale, 1975; Coxon and Pallis, 1976); the type of neuropathy is similar to that which we have encountered with Ro 07-0582. The mechanism of neurotoxicity is at present unknown. Biochemical studies concerned with the drug and its metabolic breakdown products are under way in cooperation with Roche Products Ltd.

The peripheral neuropathy was, perhaps, most troublesome in the group of 3 patients given daily doses of Ro 07-0582, and least frequent in the 5 patients given 4 doses, although the total dose in all 3 groups was similar.

A relationship between the number of administrations and neurotoxicity seemed possible; however, as a means of expressing tissue exposure to Ro 07-0582, we have calculated the areas under the curves when plasma concentration is plotted against time. We were able to

TABLE VI.—*Tissue Exposure Calculated in Arbitrary Units (see text) in the Cases Receiving* 4 *and* 6 *Doses of Ro* 07-0582, *Separated into Those with and without Neuropathy. Tissue Exposure seems Significantly Related to Neurotoxicity* ($P = 0{\cdot}03$)

| Case No. | Exposure index: Neuropathy | No neuropathy |
|---|---|---|
| C1 | 1·84 | |
| C3 | | 0·77 |
| C5 | | 1·16 |
| C6 | 1·27 | |
| C7 | 1·33 | |
| C10 | 1·04 | |
| C12 | 1·19 | |
| C16 | | 1·14 |
| C17 | 1·24 | |
| C18 | 1·31 | |
| C19 | 1·17 | |
| C21 | | 0·77 |

do this only with those cases with either 4 or 6 doses. The results expressed in arbitrary units were calculated for the cases and these were then separated into “ no toxicity ” and “ toxicity ” groups (Table VI). Tissue exposure appeared significantly related to neurotoxicity ($P = 0{\cdot}03$). It seems likely that total tissue exposure is the most significant factor, and frequency of dose less important, in the incidence of toxicity. The administration of the B vitamins did not appear to prevent neuropathy.

The estimates of tumour concentration show that the drug readily passes into the tumour, and that concentrations approaching those in plasma are achieved. It is of interest that similar high tumour concentrations have been found with the other nitroimidazole which has been tested in man as a hypoxic cell sensitizer, metronidazole (Urtasun *et al.*, 1976). Evidence has been presented to show that the drug will penetrate into tissue likely to contain hypoxic cells, and some will even reach totally necrotic debris.

The patients reported in this study all presented with advanced malignant disease. In 13 cases there was an extensive tumour in the primary site, while in 3 metastatic disease was treated. Tumour response has been followed carefully in these patients and, when assessed at 2 months after initiation of treatment, regression seemed complete in 8 and partial in another 8. These responses are, perhaps, better than might be expected with conventional radiotherapy. However, no true estimate as to the effectiveness of the drug to improve radiotherapy can be obtained from a series of uncontrolled cases of this sort. When a safe, satisfactory regime for administration of Ro 07-0582, in conjunction with radiotherapy, is established then, in controlled studies, the value of this radiosensitizer in radiotherapy can be assessed.

In all cases, careful observation was made of immediate reactions in normal tissues. The areas irradiated were in the neck, chest, lower abdomen and pelvis. All immediate reactions in the pharynx, oesophagus, lung and bowel were similar to those encountered when treatment is given without the sensitizer. As with tumour response, only a careful comparison of reactions in a randomized controlled clinical trial can give a final answer. Animal work and our study of skin reaction in man suggests that in oxygenated tissues there is no increase in radiation response (Dische *et al.*, 1976).

In recent years there has been considerable interest in multiple treatments each day with radiotherapy, and some promising results have been reported (Choi and Suit, 1975; Svodoba, 1975; Littbrand, personal communication; Douglas, personal communication). The addition of Ro 07-0582 to such regimes is particularly practical when treatment is given on a limited number of days.

In multiple-fraction studies of the radiotherapy of animal tumours, it has been shown that the addition of Ro 07-0582 to certain treatments early and/or late in a course gives a surprisingly high gain in effect (Van Putten and Smink, 1976; Sheldon, personal communication).

Using Ro 07-0582 with a single dose of radiotherapy, an enhancement of response has been observed (Thomlinson *et al.*, 1976). It is possible that, in palliative radiotherapy, single treatments using Ro 07-0582 could replace multiple-fraction techniques, giving similar palliation with greater ease for the patient and with less demand on treatment and hospital facilities.

The incidence of neurotoxicity in our patients given multiple doses of Ro 07-0582 was high, amounting to 75% (12/16). The severity was such that we must limit the amount of drug to be given in future work. We believe that a reduction of 20% in total dose from 15 g/m² to 12 g/m² may give a marked reduction in morbidity. In our next series of studies we intend to give no more than a dose of 12 g/m² or 300 mg/kg in any one course. Individual doses of Ro 07-0582 will not exceed 5 g/m² or 125 mg/kg.

There is a wide range of clinical application possible within this dose limitation. We are now exploring the use of Ro 07-0582 in a number of different ways:

(1) In a low daily dosage.

(2) Combined with all 6 fractions in a course of radiotherapy over 17 to 18 days.

(3) Employing up to 6 doses combined with multiple treatments on each day of administration.

(4) Adding to certain treatments only in a multifraction course of radiotherapy.

(5) With single doses of radiotherapy for palliation.

With the dose limitation we must consider the radiosensitization to be expected with use of these differing regimes. New data on tumour concentrations in animals and man considerably influence the situation.

In radiobiological experiments with mouse tumours where there was a large improvement in tumour control, drug dosages calculated on a mg/kg basis have been higher than those used in our patients who suffered neurotoxicity.

However, in the mouse, the clearance rate of Ro 07-0582 is comparatively rapid (1–1½ h half-life), and this is reflected in estimates of tumour concentration, rarely exceeding 40% of that in plasma. In contrast, in man where drug clearance is about 8-fold slower, our data indicate that tumour levels are similar to those in plasma. The dose required for sensitization will, on an mg/kg basis, be appreciably lower in man than in mouse.

An additional benefit of the longer half-life in man will be the loss of repair of sub-lethal injury in the hypoxic cells. This did not contribute to the improved results in the mouse tumour systems so far reported, for radiotherapy was by necessity, due to the short half-life, given within an hour of administration of Ro 07-0582.

In Fig. 5 we have shown the enhancement ratio for sensitization of hypoxic cultivated Chinese hamster cells as a function of drug concentration (data from Adams *et al.*, 1976). Also shown are the results of the irradiation of skin made temporarily hypoxic in patients given single doses of Ro 07-0582 (Dische, *et al.*, 1976). The similarity of the data supports the view that all mammalian cells will respond similarly when irradiated under hypoxia with the administration of Ro 07-0582.

The shape of the curve relating enhancement ratio to drug concentration is such that considerable enhancement is present at relatively low concentrations and, as this rises, the increase of benefit becomes proportionately less.

In our patients given daily doses of Ro 07-0582, the mean plasma concentrations lay between 32 and 43 μg/ml. Such levels correspond to enhancement ratios between 1·42 and 1·51. We believe it should be possible to achieve 20 μg/ml with acceptable morbidity, and this should give an enhancement ratio of 1·3. This calculation, like the data from the mouse

FIG. 5.—The sensitizing efficiency of Ro 07-0582. Continuous line: Data for sensitization of hypoxic Chinese hamster V79 cells cultured and irradiated *in vitro* (Adams *et al.*, 1976). ●: Data for sensitization of hypoxic human skin. (Adapted from Dische *et al.*, 1976.)

tumour systems, does not include any additional benefit which may be gained in man because of the prolonged exposure and loss of repair of sub-lethal injury sustained by hypoxic cells.

When a patient is given 6 doses of Ro 07-0582, it should be possible to reach a tumour concentration giving an enhancement ratio of 1·65: a value comparable to the theoretical single-dose gain factor predicted for neutron radiotherapy.

Work proceeds to develop other chemical hypoxic cell sensitizers which may have a more favourable ratio of sensitizing effect to morbidity. However, Ro 07-0582 shows considerable promise as a drug likely to improve the results of radiotherapy. The benefits which may occur with the introduction of an effective hypoxic cell sensitizer are great (Adams *et al.*, 1976) and there is good encouragement to continue the further testing of Ro 07-0582.

We wish to thank Dr C. H. Hassall, Dr I. Lenox-Smith, Dr C. E. Smithen and Roche Products Ltd., Welwyn Garden City, for their continued cooperation. Dr Jack Fowler and our colleagues in the Gray Laboratory have given their continued advice and encouragement, and Miss D. Troup her valued technical assistance. Dr M. H. Bennett has advised upon the pathology and Dr L. S. Lange upon neurological aspects of this work. Physics, junior medical, radiographic and nursing staff have continued to give us their close collaboration.

Our grateful thanks are due to the Medical Research Council (SD, MIS and MEL) and the Cancer Research Campaign (GEA and IRF) for their support.

### REFERENCES

ADAMS, G. E. & FOWLER, J. F. (1976) Nitroimidazoles as Hypoxic Cell Sensitizers *In vitro* and *In vivo*. In *Modification of Radiosensitivity*

*of Biological Systems.* Vienna: I.A.E.A. p. 103.

ADAMS, G. E., FLOCKHART, I. R., SMITHEN, C. E., STRATFORD, I. J., WARDMAN, P. & WATTS, M. E. (1976) Electron-affinic Sensitization VII. A Correlation Between Structures, One-electron Reduction Potentials, and Efficiencies of Nitroimidazoles as Hypoxic Cell Radiosensitizers. *Radiat. Res.*, **67**, 9.

CHOI, C. H. & SUIT, H. D. (1975) Evaluation of Rapid Radiation Treatment Schedules Utilizing Two Treatment Sessions per Day. *Radiology*, **116**, 703.

COXON, A. & PALLIS, A. (1976) Metronidazole Neuropathy. *J. Neurol. Neurosurg. Psychiatry*, **39**, 403.

DISCHE, S., GRAY, A. J. & ZANELLI, G. D. (1976) Clinical Testing of the Radiosensitizer Ro 07-0582. II. Radiosensitization of Normal and Hypoxic Skin. *Clin. Radiol.*, **27**, 159.

DISCHE, S. & SENANAYAKE, F. (1972) Radiotherapy Using Hyperbaric Oxygen in the Palliation of Carcinoma of Colon and Rectum. *Clin. Radiol.*, **23**, 512.

FOSTER, J. L., FLOCKHART, I. R., DISCHE, S., GRAY, A., LENOX-SMITH, I. & SMITHEN, C. E. (1975) Serum Concentration Measurements in Man of the Radiosensitizer Ro 07-0582: Some Preliminary Results. *Br. J. Cancer*, **31**, 679.

GRAY, A. J., DISCHE, S., ADAMS, G. E., FLOCKHART, I. R. & FOSTER, J. L. (1976) Clinical Testing of the Radiosensitizer Ro 07-0582. I. Dose Tolerance, Serum and Tumour Concentration. *Clin. Radiol.*, **27**, 151.

INGHAM, H. R., SELKON, J. B. & HALE, J. H. (1975) The Antibacterial Activity of Metronidazole. *J. Antimicrob. Chemother.*, **1**, 355.

JOHNSON, R., GOMER, C., AMBRUS, J., PEARCE, J. & BOYLE, D. (1976) An Investigation of the Pharmacological and Radiosensitizing Effects of the 2-Nitroimidazole Ro 07-0582 in Primates. *Br. J. Radiol.*, **49**, 294.

SCHÄRER, K. (1972) Selective Alteration of Purkinje Cells in the Dog after Oral Administration of High Doses of Nitroimidazole Derivatives. *Verh. dt. Ges. Pathol.*, **56**, 407.

SVODOBA, V. H. J. (1975) Radiotherapy by Several Sessions a Day. *Br. J. Radiol.*, **48**, 131.

THOMLINSON, R. H., DISCHE, S., GRAY, A. J. & ERRINGTON, L. M. (1976) Clinical Testing of the Radiosensitizer Ro. 07-0582. III. Response of Tumours. *Clin. Radiol.*, **27**, 167.

URTASUN, R. C., BOND, P., CHAPMAN, J. D., FELDSTEIN, M. L., MIELKE, B. & FRYER, C. (1976) Radiation and High Dose Metronidazole (Flagyl) in Supratentorial Glioblastomas. *New Engl. J. Med.*, **293**, 1364.

VAN PUTTEN, L. M. & SMINK, T. (1976) Effect of Ro 07-0582 and Radiation on a Poorly Reoxygenating Mouse Osteosarcoma. In *Modification of Radiosensitivity of Biological Systems.* Vienna: I.A.E.A. p. 179.

WORLD HEALTH ORGANIZATION (1963) The Prevention and Treatment of Isoniazid Toxicity in the Therapy of Pulmonary Tuberculosis. II. An Assessment of the Prophylactic Effect of Pyridoxine in Low Dosage. *Bull. Wld. Hlth. Org.*, **29**, 457.